# The roles of extracellular vesicles in major depressive disorder

**DOI:** 10.3389/fpsyt.2023.1138110

**Published:** 2023-03-09

**Authors:** Ying Li, Yan Gui, Miaomiao Zhao, Xuanqiang Chen, Haimei Li, Chen Tian, Haoyang Zhao, Chaonan Jiang, Pengfeng Xu, Shiyi Zhang, Shaoyong Ye, Manli Huang

**Affiliations:** ^1^Department of Psychiatry, The First Affiliated Hospital, Zhejiang University School of Medicine, Hangzhou, China; ^2^Key Laboratory of Mental Disorder's Management of Zhejiang Province, Hangzhou, China; ^3^Brain Research Institute, Zhejiang University, Hangzhou, China; ^4^Zhejiang Engineering Center for Mathematical Mental Health, Hangzhou, China; ^5^Department of Psychiatry, Tongde Hospital of Zhejiang Province, Mental Health Center of Zhejiang Province, Hangzhou, China; ^6^Zhejiang University School of Medicine, Zhejiang University, Hangzhou, China; ^7^Henan University School of Medicine, Henan University, Kaifeng, China

**Keywords:** major depression disorder, extracellular vesicles, microRNAs, biomarker, therapeutic carrier, neurogenesis, neuro-inflammation

## Abstract

Major depressive disorder (MDD) is a serious mental disease characterized by depressed mood, loss of interest and suicidal ideation. Its rising prevalence has rendered MDD one of the largest contributors to the global disease burden. However, its pathophysiological mechanism is still unclear, and reliable biomarkers are lacking. Extracellular vesicles (EVs) are widely considered important mediators of intercellular communication, playing an important role in many physiological and pathological processes. Most preclinical studies focus on the related proteins and microRNAs in EVs, which can regulate energy metabolism, neurogenesis, neuro-inflammation and other pathophysiological processes in the development of MDD. The purpose of this review is to describe the current research progress of EVs in MDD and highlight their potential roles as biomarkers, therapeutic indicators and drug delivery carriers for the treatment of MDD.

## 1. Introduction

### 1.1. Major depressive disorder

Major depressive disorder (MDD) is one of the most prevalent and disabling mental disorders and is characterized by depressed mood, loss of interest and suicidal ideation. Moreover, the sociality and quality of life of MDD patients are seriously impaired. MDD often reoccurs, which increases morbidity and mortality. The prevalence of MDD varies greatly in different countries and regions (1), but the global incidence is increasing every year. The lifetime prevalence of depression in Chinese adults is 6.8%, of which the prevalence of MDD is 3.4% (2). The lifetime prevalence of MDD in American adults was 20.6% (3), and in European adults, it was 11.3% (4). In 2008, the WHO listed MDD as the third largest contributor to the global disease burden and predicted that the disease would rank first by 2030. At present, the Diagnostic and Statistical Manual of Mental Disorders (DSM-5) and the 11^th^ Revision of the International Classification of Diseases (ICD-11) are regarded as the gold standard for the diagnosis of MDD. However, the symptoms of different neuropsychiatric diseases overlap to a certain extent and there is a lack of specific MDD biomarkers for accurate diagnosis and evaluation, which may lead to certain bias and misdiagnosis, thus affecting the progression and prognosis of the disease. Although more than half of MDD patients recovered within 6 months and nearly three quarters recovered within 1 year after treatment, 27.8% of MDD patients did not recover and developed chronic depression (5). Therefore, it is urgent to further explore new biomarkers of MDD to achieve early detection, diagnosis and treatment.

Researchers are currently looking for biomarkers of MDD in many directions such as neuroimaging, immunology, neurotransmitters, oxidative stress, and gastrointestinal factors. The study of structural and functional brain abnormalities in MDD patients has revealed many neuroimaging features of MDD patients (6–8). Although neuroimaging biomarkers are more stable than peripheral biomarkers, the heterogeneity of patients and differences in imaging analysis methods have led to inconsistent results across studies. C-Reactive Protein, Interleukin-6 (IL-6), IL-1ß, Tumor Necrosis Factor-α (TNFα) are considered to be common markers of inflammation (9, 10). Neurotrophic factor family:brain-derived neurotrophic (BDNF) and Glial cell line-derived neurotrophic factor (GDNF) (11, 12)，as well as oxidative stress (13) and mitochondrial metabolism-related products (14) are also potential biomarkers of MDD. Although many candidate metabolite indicators associated with depression and drug response have been identified, few of them have been validated in larger population-based cohorts, and thus there are still no biomarkers that can be applied to the clinical diagnosis and treatment of depression. Therefore, we need to continuously explore new candidate indicators and integrate them for validation.

### 1.2. Extracellular vesicles

**Figure 1:**
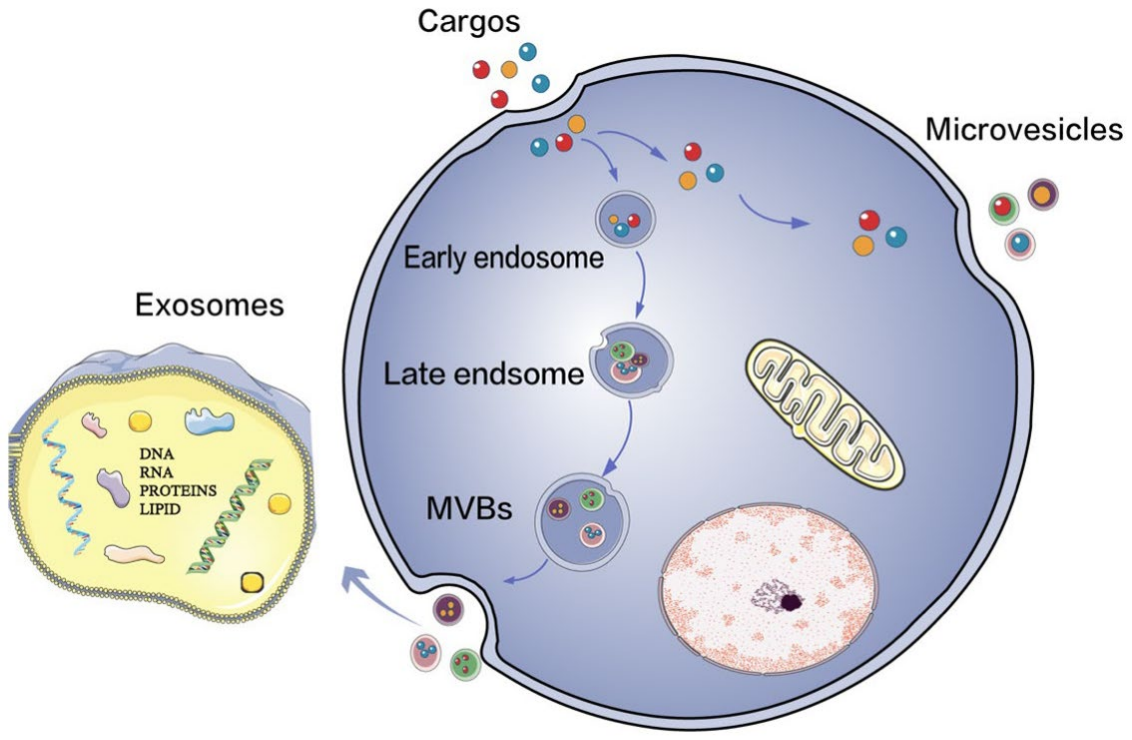
The biogenesis of EVs. Extracellular vesicles (EVs) mainly consist of two types: exosomes and microvesicles. Microvesicles bud off directly from the plasma membrane, and the production of exosomes involves the formation of multivesicular bodies (MVBs) upon transport and fusion with the plasma membrane. Their released internal vessels (ILVs) become exosomes.

Extracellular vesicles (EVs) play an important role in intercellular communication in numerous physiological and pathological processes. EVs are nanoscale vesicles that are naturally released from cells and are surrounded by a lipid bilayer. They cannot replicate because they do not have functional nuclei (15). EVs secreted by different cell-types have different components, including various proteins, lipids, nucleic acids, particle structures and other biological molecules from the cell-type they are secreted from. They act on target cells through short-distance and long-distance transport of materials and release contents to conduct signal transduction, thus regulating the functional state of target cells. Recently, extensive studies have explored the biogenesis and release of EVs, as well as the interaction with and uptake by target cells (16–20). According to the latest research on the biogenetic mechanism of extracellular vesicles (18, 20), there are two main subtypes of extracellular vesicles, namely, exosomes and microvesicles. The microvesicles germinate directly from the cell membrane, while the biogenesis of exosomes involves the formation of multivesicular bodies (MVBs) (Figure 1). However, due to the partial overlap between the two, existing technologies cannot distinguish the subtypes well, and no consensus has been reached on the specific markers of the subtypes of EVs. According to the most recent guidelines of the International Society of Extracellular Vesicles (ISEV), the general term “EVs” is used in this review to summarize exosomes, multivesicular bodies and other terms to avoid misunderstanding or incorrect definitions.

Almost all types of cells can secrete EVs, and EVs are abundant and stable in biological fluids such as plasma and urine. As nanoscale particles, EVs can easily overcome various biological barriers, the most attractive of which is their compatibility with the blood–brain barrier. These characteristics make them promising as new biomarkers of diseases and therapeutic drug delivery carriers. The molecules carried on the surface of EVs can better distinguish their origin, giving us a clearer picture of the changes in the brain. The potential role of EVs in the aetiology and pathophysiology of psychiatric and neurodegenerative diseases has received great attention (21–24). Accumulating evidence shows that EVs are the bridge of intercellular signal transduction and exchange of materials within the local brain regions, the brain as a whole and the periphery.

### 1.3. The intersection of EVs and MDD

**Figure 2:**
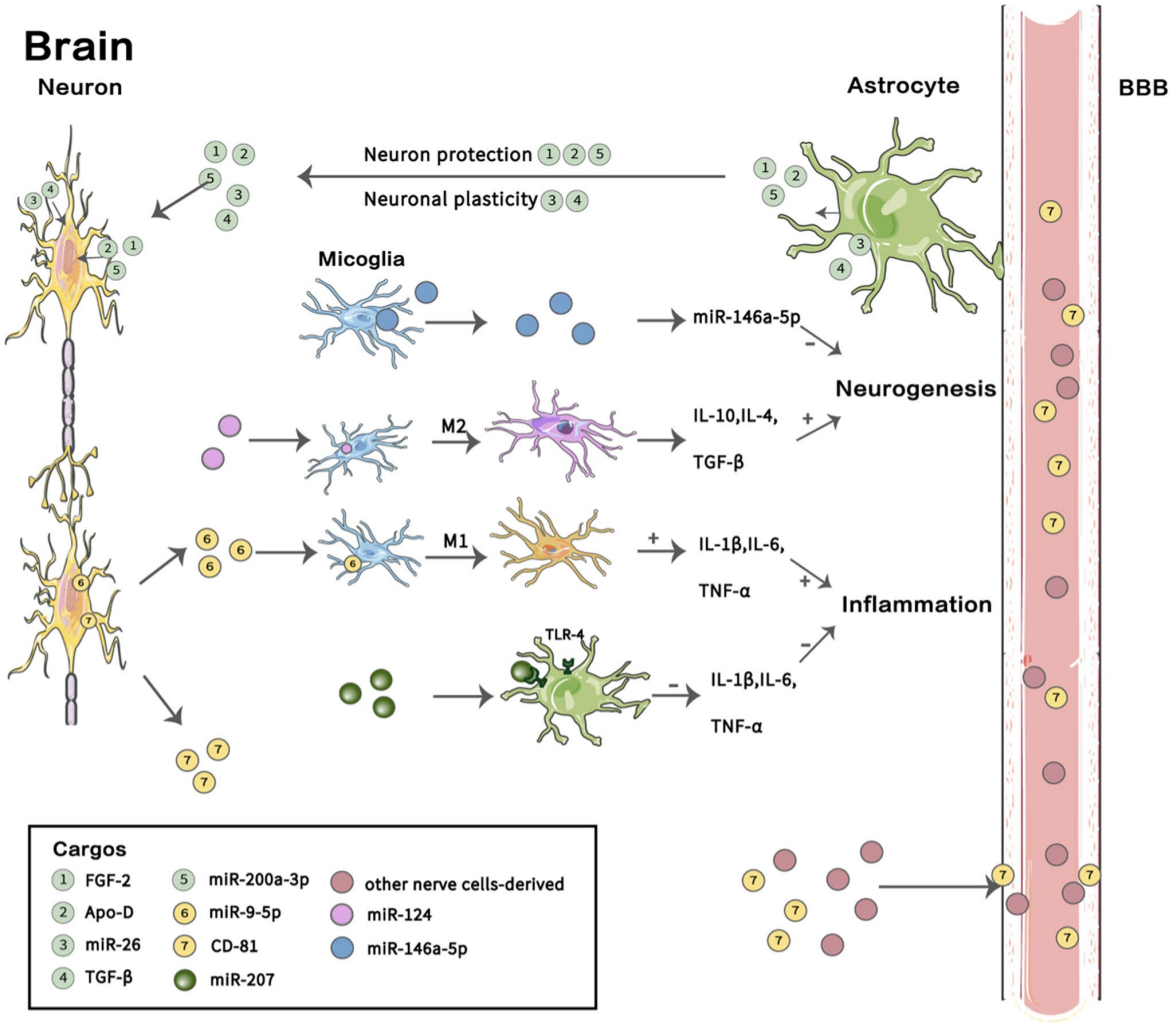
Cargos in EVs involved in the pathogenesis of MDD. In the brain, astrocytes secrete extracellular vesicles (EVs) containing FGF-2, Apo-D, and miR-200a-3P that act on neurons and participate in neuroprotection under oxidative stress, and they also secrete EVs containing miR-26 and TGF-β, which play a role in neural plasticity. EVs containing miR-207 secreted by NK cells act on the TLR-4 receptor of astrocytes and inhibit the release of IL-1 β, IL-6 and TNF-α to reduce neuro-inflammation. Microglia undergo M1 polarization and M2 polarization, thus participating in different pathophysiological processes. EVs secreted by microglia containing miR-146a-5P can inhibit neurogenesis. EVs with miR-9-5P promote M1 microglia polarization and release IL-4, IL-10 and TGF-β to support neurogenesis; however, EVs containing miR-26 induce M2 polarization of microglia and release the pro-inflammatory factors IL-1β, IL-6 and TNF-α to promote inflammation. Brain-derived EVs transfer to the periphery through the blood–brain barrier (BBB), and abnormal expression of some proteins and miRNAs was found in neuron-derived EVs in plasma. A summary of several previous findings described in this review is shown. BBB, blood–brain barrier; FGF-2, Fibroblast growth factor 2；Apo-D, Apolipoprotein D; TGF-β, transforming growth factor-β. Images for these figures were adapted from Servier Medical Art (by Servier; https://smart.servier.com/), licenced under CC BY 3.0 (https://creativecommons.org/licenses/by/3.0/).

The pathogenesis of MDD mainly involves dysfunction of the hypothalamic pituitary adrenal (HPA) axis, neurotransmitter metabolism disorder, synaptic plasticity, oxidative stress, intestinal flora and neuroinflammation (25, 26) and other pathological processes. Current research has found that EVs and their contents that are associated with MDD are also mainly involved in energy metabolism, neuro-inflammation, and neurogenesis (27–33) and other pathogenic mechanisms (Figure 2). Most preclinical studies focus on the related proteins and RNAs of EVs. The main type of RNA in EVs is microRNA (miRNA), with an average length of 22 nucleotides. MiRNAs have complex regulatory networks, which can not only shuttle between different cells to control the rate of translation and transcription (25, 26)but also act as a signal molecule to mediate intercellular communication (27–30). RNA sequencing showed that miRNAs were highly enriched in EVs, and different physiological conditions of donor cells, such as oxidative stress (31, 33), exercise (32), and pain (33) may lead to the presence of specific miRNAs (34). The proteins and miRNAs in EVs play a key role in the physiological and pathological processes of MDD. Many proteomic and genealogical studies have shown that the proteins (29, 35) and miRNAs (30, 36, 37) carried in EVs are very likely to be biological diagnostic markers of depression. This review will describe the proteins and RNAs carried by EVs in patients with depression or depression animal models that are related to pathophysiological processes such as energy metabolism, neuro-inflammation, neurogenesis, and the blood–brain barrier (BBB) and discuss their potential as biological diagnostic markers, therapeutic indicators, and drug delivery carriers for the treatment of MDD.

## 2. Methods

### 2.1. Search strategy and study selection

All articles included in this review were found through the PubMed database and Google Scholar. The search deadline was July 2022. The following search keyword combinations were used: (“depression disorder” or “major depressive disorder”), (“microRNA” or “miR”), (“extracellular vesicles” or “EVs”), (“exosomes” or “ectosomes”), (“mental disorder” or “central nervous system diseases”), (“brain gut axis” AND “major depressive disorder”), (“blood–brain barrier” AND “major depressive disorder”), (“neurogenesis” AND “major depressive disorder”), (“neuro-inflammation” AND “major depressive disorder”), and (“protein” AND “extracellular vesicle” AND “major depressive disorder”). Each combination of search terms produced an important list of research in which we selected articles that were consistent with our goals and interests.

After the electronic search, duplicate references were excluded. Titles, abstracts, and study methodologies were screened based on the inclusion and exclusion criteria.

### 2.2. Inclusion and exclusion criteria

Research articles’ inclusion criteria were: (1) Studies on the direction of extracellular vesicles in MDD patients and animal models, (2) Since studies doing extracellular vesicles in the direction of major depressive disorder are more limited, we targeted all subtypes and sizes of extracellular vesicles as a way to obtain more comprehensive information, (3) Published in the English language.
Research articles’ exclusion criteria were: (1) Studies of bipolar disorder and other non-major depressive disorders, (2) Non-extracellular vesicles oriented researches, (3) Duplicate studies, and (4) Papers not written in the English language.

## 3. Potential role of extracellular vesicles in major depressive disorder

### 3.1. Extracellular vesicles are involved in energy metabolism

In recent years, the dysfunction of energy metabolism in mitochondria, the accompanying oxidative stress and rise of reactive oxygen species (ROS) have increasingly been regarded as one of the signs of MDD pathophysiology. Usually, MDD patients exhibit individual differences regarding sensitivity to stress, even under the same stressful conditions. The morphology and metabolism of mitochondria, as well as their distribution and transport, are crucial for the ability to cope with stress (38). In recent years, the intercellular transfer of mitochondria and mitochondrial components through EVs has been a topic of research (39, 40). Mitochondrial DNA (mtDNA) released from damaged mitochondria can be packaged into EVs generated by mitochondria for transfer, and activate NF-κB, interferon regulatory factor 1 (IRF-1) and caspase-1 activated by interacting with Toll-like receptors (TLRs). The cytosolic cGAS-STING DNA sensing system and the NLR family pyrin domain containing 3 (NLRP3) inflammasome can stimulate proinflammatory pathways (40, 41). A recent study extracted and detected mitochondrial proteins in EVs derived from neurons in the plasma of MDD patients. It was found that the mitochondrial functional proteins in neuron-derived EVs (NDEVs) of MDD patients were reduced and widely normalized after SSRI treatment. These proteins are involved in mitochondrial biogenesis, mitochondrial dynamics and function maintenance (TFAM, CYPD, MFN2, SNPH, MY06), mitochondrial energy metabolism (NMNAT2, SARM1), neuroprotection (human), and regulation of neuronal metabolism (MOTS-c) (14). In addition, insulin signal transduction is crucial for systemic energy metabolism. Some studies have shown that the level of insulin receptor substrate 1 (IRS-1) in plasma NDEVs of MDD patients is increased and is related to suicide and loss of pleasure (35). In normal physiological conditions, EVs secreted by astrocytes transport apolipoprotein D (ApoD) to neurons to participate in neuroprotection under oxidative stress (42). Furthermore, EVs containing fibroblast growth factor-2 (FGF-2) secreted by astrocytes help cells survive under oxidative stress by reducing ER stress and mitochondrial damage (43). Therefore, we hope to reflect energy metabolism in the brain by detecting energy metabolism indicators such as mitochondrial-related proteins and IRS-1 in plasma brain-derived EVs, which provides a new idea for us to explore the energy metabolism mechanism of depression. After treatment, the levels of mitochondrial proteins in corresponding EVs return to normal, and these proteins could likely to be used as a therapeutic indicator of depression. Since EVs can successfully pass the blood–brain barrier, they can be considered transport carriers to transport ApoD, FGF-2 and other neuroprotective factors and drugs for precise treatment in the brain.

### 3.2. Extracellular vesicles are involved in neuro-inflammation

There has been growing evidence that MDD is associated with systemic immune activation (44–47). In patients with MDD, the level of inflammatory mediators including cytokines, chemokines and other various inflammatory mediators, increases in the peripheral and central nervous system A meta-analysis showed that peripheral levels of interleukin-6 (IL-6), tumour necrosis factor (TNF)-alpha, IL-10, the soluble IL-2 receptor, C-C motif chemokine ligand 2 (CCL2), IL-13, IL-18, IL-12, the IL-1 receptor antagonist (IL-1RA), and the soluble TNF receptor 2 were elevated in patients with MDD compared to those in healthy controls, whereas interferon-gamma levels were lower in MDD patients (48). EVs may be involved in the process of neuro-inflammation in MDD patients (49), and many researchers have explored inflammatory markers in NDEVs in blood to identify objective biological indicators of MDD. In one study, NDEVs were standardized through detection of CD81 expression, and researchers detected that IL-34, synaptophysin (SYP), tumour necrosis factor receptor 1 (TNFR1) and CD81 were co-expressed. This means that brain-derived IL-34, SYP, and TNFR1 might be related to depression-like behaviour and depression symptoms. Moreover, IL-34 may be a biological diagnostic marker of depression (50). In addition, sigma-1 receptor (Sig-1R) is an upstream regulator of ER stress and regulates ER-mitochondria signaling and ER-nucleus crosstalk (51). Sig-1R can regulate the bioenergetics and oxidative stress of mitochondria (52). It has also been proven to be significantly enriched in EVs from depression animal models and patients. Plasma EVs from depression patients improve depressive behaviour induced by inflammation through the transmission of Sig-1R, and the injection of EVs from depression model mice or depression patients significantly improves the reduced BDNF expression, neuro-inflammation, and depressive behaviour of mice stimulated with lipopolysaccharide (34, 53). Microglia are special immune cells in the brain and account for 5–10% of all brain cells. Recent studies have shown that microglia are activated in many neurodegenerative and neuropsychiatric diseases, and promote the occurrence of neuroinflammation (54). In MDD patients, miR-9-5p is transferred from neurons to microglia through EVs, leading to the polarization of M1 microglia and excessive release of proinflammatory factors, thereby damaging neurons (55). In addition, activated microglia secrete the cytokine interleukin-1 β (IL-1 β), the IL-1 β-processing enzyme caspase-1, and P2X7, thus promoting the occurrence and diffusion of neuro-inflammation in the brain. A study showed that NK cell-derived EVs that carry miR-207 reduce the release of proinflammatory cytokines, miR-207 directly targets TLR4 interactor with leucine-rich repeats (Tril), and inhibits NF-κB signal transduction in astrocytes to suppress depression-like symptoms in mice (56).

### 3.3. Extracellular vesicles are involved in neurogenesis and neuroplasticity

Neurogenesis is an inherent physiological process that depends on the existence and maintenance of neural stem cells and neural progenitor cells. From the proliferation of neural stem cells to the maturation and survival of neurons, each step is strictly regulated by multiple signaling factors in the local microenvironment (57, 58). Abnormalities in neurogenesis cause a series of mental disorders (59–61). Researchers have proposed two hypotheses to explain the decrease in hippocampal volume in MDD patients: (i) the neuroplasticity hypothesis and (ii) the neurogenesis hypothesis (62). As a new regulator of the adult neurogenic microenvironment, EVs may participate in neurogenesis and synaptic plasticity(63). The miRNAs’ expression profile in plasma NDEVs of depressed patients is abnormal (37), and the dysregulated miRNAs mainly affect postsynaptic density, axon formation and signaling pathways of cell growth (64). The levels of miR-139-5p were upregulated in serum NDEVs in MDD patients, and could be used as a potential biomarker for the diagnosis of MDD (65). Another study found that miR-139-5p upregulation was the highest differential expression. It was also proven that the increased level of miR-139-5p in exosomes may mediate stress-induced depression-like behaviour in mice by negatively regulating the proliferation of neural stem cells and neuronal differentiation (66). A sequencing analysis of miRNAs in plasma exosomes of patients with refractory depression found that there were significant differences in the expression of two miRNAs, the upregulated miR-335-5p and the downregulated miR-1,292-3p. They also carried out target gene Gene Ontology (GO) enrichment analysis and Kyoto Encylcopedia of Genes and Genomes (KEGG) pathway enrichment analysis and found that the dysfunctional miRNAs affect postsynaptic density and axonal formation. In addition, microglia inhibit neurogenesis in MDD patients by secreting EVs rich in miR-146a-5p, and miR-146a-5p inhibits neurogenesis and spontaneous discharge of excitatory neurons by directly targeting Krüppel-like factor 4 (KLF4) (67). Moreover, EVs rich in miR-124 can promote the polarization of M2 microglia and enhance hippocampal neurogenesis (68). M2 microglia secrete the anti-inflammatory cytokines IL-4, IL-10 and TGF-β, which are beneficial to hippocampal neurogenesis. In addition, astrocytes play an important role in maintaining the structure and function of the central nervous system. The miRNAs in astrocyte-derived EVs act as regulators of adult neurogenesis and stress responses (69). For example, miR-26 affects the function and morphology of neurons as a possible mediator of neuronal plasticity (70). Normal astrocytes release EVs containing miR-200a-3p, which shows neuroprotective effects by downregulating MKK4 (71). In addition, astrocyte EVs mediate TGF-β signal activation through fibulin-2, which promotes synapse formation (72). In conclusion, the miRNAs in EVs participate in the signal regulation of neurogenesis, neuroprotection, and synapse formation and have the potential to be biomarkers of MDD.

### 3.4. Extracellular vesicles are involved in the blood–brain barrier and intestinal mucosal barrier

The blood–brain barrier (BBB) is a complex barrier system with high selectivity and is composed of neurovascular units (NVUs) consisting of endothelial cells, pericytes, astrocytes, microglia and neurons (73, 74). In addition to the aforementioned energy metabolism, neuro-inflammation, neurogenesis and synaptic plasticity, EVs also participate in other pathophysiological processes of MDD pathogenesis. In recent years, BBB dysfunction has become a new hypothesis for the pathogenesis of MDD (75–77). EVs are closely related to the function of the blood brain barrier itself, and may mediate the maintenance and regulation of BBB permeability (78). FGF-2 and vascular endothelial growth factor (VEGF) belong to the growth factor family. There is evidence that astrocytes secrete EVs containing FGF-2, which are conducive to maintaining the integrity of the blood–brain barrier (BBB) (43). In addition, VEGF/VEGFR2 play key roles in the pathogenesis of depression by increasing BBB permeability (79). Whether this role depends on EVs mediation needs further verification. In patients with MDD, endothelial cell dysfunction, increased permeability of the BBB (4–7) (80–83), and BBB dysfunction may lead to peripheral substance infiltration and increased EVs’ passage. A study on stress and emotional disorders showed that the increase in the concentration of EVs derived from astrocytes in peripheral blood may be due to the increased permeability of the BBB (84). Some studies have shown that antidepressants can promote cerebral angiogenesis and brain tissue remodeling and increase the integrity of the BBB. Their role in promoting angiogenesis is mediated by small EVs released by endothelial cells. The uptake of these vesicles in endothelial cells increases, thus increasing the integrity of the blood–brain barrier, reducing leukocyte infiltration, and increasing neuronal survival (85). Therefore, EVs may also participate in the pathogenesis of MDD by maintaining and regulating the function of the BBB. Besides the BBB, intestinal barrier dysfunction is accompanied by inflammation in MDD patients, and the degree of intestinal barrier dysfunction is related to the severity of MDD symptoms (86). EVs produced by host and intestinal microorganisms affect intestinal mucosal barrier function together (87–89)and have become an important part of bacterial-host communications (90). Gut-brain axis (GBA) dysfunction is also a pathogenic characteristic of MDD, and may lead to subclinical inflammation, hypothalamic pituitary (HPA) axis imbalance, and changes in neural, metabolic and endocrine pathways (90, 91). In mice, researchers found that Lactobacillus-derived EVs can change the expression of BDNF in the hippocampus and exert antidepressant-like effects in stress-induced depression model mice (92). Moreover, EVs of mouse intestinal flora can affect the expression of inflammatory factors and regulate the signal transduction and metabolism of serotonin through the GBA (93).

## 4. Discussion

This review summarizes the current research progress of the role of EVs in MDD, mainly involving energy metabolism, neuro-inflammation, neurogenesis and neuroplasticity pathways. EVs carry the bioactive components of secretory cells, transport them to target cells and then release the contents for material exchange and signal transduction. Differential ultracentrifugation, density gradients, precipitation, filtration, size exclusion chromatography and immunoisolation are often used to capture EVs in the circulatory system, detect their contents and characteristics, and trace their source. The newly developed nanotechnology (94, 95)and biosensor platform (96) can detect EVs more efficiently and sensitively, and there are also many new methods (97, 98)for the detection of contents of EVs. With the development of new techniques, we can detect and measure EVs secreted by the CNS in the periphery. These EVs can reflect the pathophysiological changes of the CNS to a certain extent, giving them potential as biomarkers of MDD. Some researchers compared EVs between depression patients or depression model animals and healthy controls and found many differentially expressed miRNAs (55, 66, 99, 100)and proteins (Table 1). These differentially expressed proteins and miRNAs may be biomarkers of MDD and help us further explore the pathophysiological mechanism of MDD. However, because there is still no standardized extraction protocol among the subtypes of EVs, the abundance of these subtypes can make a large difference in analyses, and currently this distinction cannot be compared across platforms (101). Some of these findings remain debatable due to the inconsistent sample sources of the various studies, for example, some investigators extracted EVs from plasma and others from serum, while a recent study showed that platelets release large amounts of EVs during coagulation (102), suggesting that the results of these experiments need further discussion. In the future, it is still necessary to further clarify the specificity of the subtypes of EVs and the consistency of enrichment methods on various platforms. Rigorous selection of sample sources is also critical. In general, the proteins and miRNAs associated with EVs are still very promising MDD biological diagnostic markers.

**Table 1:** Differentially expressed EVs’ proteins in MDD subjects versus matched healthy controls and stress model rats versus control group.

Proteins	Model/disease	Species	Sample source	Expression	Reference
Mitofusin-2	MDD	Human	NDEVs	↓	Goetzl et al. (14)
Cyclophilin D	MDD	Human	NDEVs	↓	Goetzl et al. (14)
Syntaphilin	MDD	Human	NDEVs	↓	Goetzl et al. (14)
Myosin VI	MDD	Human	NDEVs	↓	Goetzl et al. (14)
LETM1	MDD	Human	NDEVs	↓	Goetzl et al. (14)
COMPLEX I-6	MDD	Human	NDEVs	↓	Goetzl et al. (14)
Complex III-10	MDD	Human	NDEVs	↓	Goetzl et al. (14)
NMNAT2	MDD	Human	NDEVs	↓	Goetzl et al. (14)
SARM 1	MDD	Human	NDEVs	↑	Goetzl et al. (14)
Humanin	MDD	Human	NDEVs	↓	Goetzl et al. (14)
MOTS-c	MDD	Human	NDEVs	↓	Goetzl et al. (14)
NRF2	MDD	Human	NDEVs	↓	Goetzl et al. (14)
IRS-1	MDD	Human	NDEVs	↑	Nasca et al. (35)
Synaptophysin	stress model	Rat	EVs in plasma	↓	Gómez-Molina et al. (36)
Reelin	stress model	Rat	EVs in plasma	↓	Gómez-Molina et al. (36)
Aldolase C	stress model	Rat	EVs in plasma	Restraint: ↑ Immobilization: ↓	Gómez-Molina et al. (36)
Astrocytic GFAP	stress model	Rat	EVs in plasma	Restraint: ↑ Immobilization: ↓	Gómez-Molina et al. (36)
IL-34	MDD	Human	EVs in plasma	↑	Kuwano et al. (50)
Sigma-1 receptor	MDD	Human	EVs in plasma	↑	Wang et al. (34)
SERPINF1	MDD	Human	EVs in plasma	↓	Jiang et al. (99)

EVs, Extracellular Vesicles; NDEVs, neuron-derived extracellular vesicles; LETM1, leucine zipper EF-hand containing transmembrane 1 protein; NMNAT2, nicotinamide mononucleotide adenylyltransferase 2; SARM1, Sterile Alpha and TIR motif-containing protein 1; MOTS-c, mitochondrial open-reading frame of the 12S rRNA-c; NRF2, transcriptional type 2 nuclear respiratory factor; IRS-1, insulin receptor substrate 1; GFAP, glial fibrillary acid protein.

The contents of EVs changed significantly before and after antidepressant treatment (Table 2). Researchers compared the changes in EVs in the plasma of MDD patients before and after antidepressant treatment. As mentioned above, mitochondrial-related proteins in brain-derived EVs of patients are widely abnormal but generally normalize after antidepressant treatment. A study evaluated the changes of BDNF/pro-BDNF levels during treatment and their correlation with clinical improvement in patients with MDD. Before antidepressant treatment, BDNF in patients’ plasma EVs was significantly reduced compared with that of the control group, while the pro-BDNF levels were the opposite. However, there was no significant difference between the two groups after 7 weeks of treatment (103). The BDNF levels before and after treatment did not follow the expected trend, which may be because the source of EVs was not determined in this study, and the same mechanism may also be involved in the periphery. Moreover, some studies have explored how miRNAs change during antidepressant treatment and found that miR-423-3p, miR-191-5p, miR-486-5p, miR-30d-5p, miR-425-5p, miR-25-3p, miR-21-5p, miR335-5p, and miR-126-5p changed significantly during antidepressant treatment. Then, stepwise regression analysis found that the combination of miR-21-5p, miR-30d-5p, and miR-486-5p changed over antidepressant treatment and was associated with the antidepressant treatment response (104).

**Table 2:** Changes of proteins in EVs before and after antidepressant treatment.

EVs contents	Source	Species	Before	After	Reference
Mitofusin-2	MDD NDEVs	Human	↓	Normalization	Goetzl et al. (14)
Cyclophilin D	MDD NDEVs	Human	↓	Normalization	Goetzl et al. (14)
Syntaphilin	MDD NDEVs	Human	↓	Normalization	Goetzl et al. (14)
Myosin VI	MDD NDEVs	Human	↓	Normalization	Goetzl et al. (14)
LETM1	MDD NDEVs	Human	↓	Normalization	Goetzl et al. (14)
Complex III-10	MDD NDEVs	Human	↓	Normalization	Goetzl et al. (14)
NMNAT2	MDD NDEVs	Human	↓	Normalization	Goetzl et al. (14)
SARM 1	MDD NDEVs	Human	↑	Normalization	Goetzl et al. (14)
Humanin	MDD NDEVs	Human	↓	Normalization	Goetzl et al. (14)
MOTS-c	MDD NDEVs	Human	↓	Normalization	Goetzl et al. (14)
BDNF	MDD serum EVs	Human	↓	Normalization	Gelle et al. (103)
proBDNF	MDD serum EVs	Human	↑	Normalization	Gelle et al. (103)
miR-423-3p	MDD NDEVs	Human	Coefficient of expression with ΔMADRS: −0.0646; *p* = 0.004		Saeedi et al. (104)
miR-191-5p	MDD NDEVs	Human	Coefficient of expression with ΔMADRS: −0.032; *p* = 0.01		Saeedi et al. (104)
miR-486-5p	MDD NDEVs	Human	Coefficient of expression with ΔMADRS: −0.0389; *p* = 0.021		Saeedi et al. (104)
miR-30d-5p	MDD NDEVs	Human	Coefficient of expression with ΔMADRS: −0.0472; *p* = 0.035		Saeedi et al. (104)
miR-425-5p	MDD NDEVs	Human	Coefficient of expression with ΔMADRS: −0.0298; *p* = 0.043		Saeedi et al. (104)
miR-25-3p	MDD NDEVs	Human	coefficient of expression with ΔMADRS: −0.0305; *p* = 0.051		Saeedi et al. (104)
miR-126-5p	MDD NDEVs	Human	Coefficient of expression with ΔMADRS: −0.0351; *p* = 0.074		Saeedi et al. (104)
miR-21-5p	MDD NDEVs	Human	Coefficient of expression with ΔMADRS: −0.0232; *p* = 0.067		Saeedi et al. (104)
miR335-5p	MDD NDEVs	Human	Coefficient of expression with ΔMADRS: −0.0351; p = 0.074		Saeedi et al. (104)

And logistic regression analysis of MDD cases demonstrated that nine miRNAs were altered across time as represented by a change in MADRS score (*p* value with sandwich correction <0.1). NDEVs, neuron-derived extracellular vesicles; LETM1, leucine zipper EF-hand containing transmembrane 1 protein; NMNAT2, nicotinamide mononucleotide adenylytransferase 2; SARM1, Sterile Alpha and TIR motif-containing protein 1; MOTS-c, mitochondrial open-reading frame of the 12S rRNA-c; BDNF, Brain Derived Neurotrophic Factor.

Because of the strict selective permeability of the BBB, there are great obstacles in the treatment of CNS diseases. EVs can easily cross the blood–brain barrier and are a bridge for the exchange of central and peripheral substances, making them a potential new carrier for targeted treatment CNS diseases (97, 105–107). Moreover, CircDYM is a homologous circular RNA (circRNA) found in humans and mice. Targeting EVs containing circDYM in the brain can reduce the depression-like behaviour induced by chronic unpredictable stress (CUS). Studies have found that circDYM EVs significantly inhibit microglial activation, BBB leakage and peripheral immune cell infiltration and alleviate the dysfunction of astrocytes induced by CUS (108). Moreover, EVs may also mediate the treatment of depression and other CNS diseases through Chinese herbal medicine (109). Researchers obtained EVs secreted by NK cells through the *in vitro* culture of NK cells. Before injecting them into the body, they carried out fluorescent labelling, injected the labelled EVs into the chronic mild stress (CMS) model mice, and then conducted behavioural tests a week later. The results not only showed that EVs from NK cells could pass through the BBB and target astrocytes but also illustrated that *in vitro* injection of EVs containing miR-207 from NK cells could reduce the depressive behaviour of mice (56). Many studies have also injected EVs with specific contents to achieve an antidepressant effect in vivo (34, 55, 66). These findings help us to understand the role of EVs and explore how to achieve systemic drug delivery or target corresponding sites in the brain through EVs, which opens a new avenue for the treatment of MDD.

## 5. Conclusion

As a mediator of intercellular communication, EVs play an important role in the physiological and pathological processes of MDD. This review describes the potential of EVs as new biomarkers of MDD as EVs affect energy metabolism, neuro-inflammation, and neurogenesis processes in the development in MDD, and are conducive to finding new targets for MDD treatment. Because of their suitable biocompatibility, low immunogenicity, stability and ability to cross the BBB, EVs may become promising drug delivery carriers for new treatments of MDD. Most studies to date have focused on the exploration of related proteins and miRNAs in EVs. There are many studies on lipids in other CNS diseases, but there is lack of evidence of related lipids in EVs of MDD patients. Despite some disputes regarding the definition and detection of EVs, there is no doubt that EVs have broad prospects in the future diagnosis and treatment of MDD. Future studies are needed to evaluate the relevant role of EVs in the pathogenesis of MDD in-depth, explore more accurate detection methods, investigate bioactive lipids in EVs of MDD patients and their roles, and develop new EV-based treatment strategies for MDD.

## Author contributions

YL: conceptualization, software, resources, and writing—original draft preparation. YL and YG: methodology. YL, YG, and MZ: visualization. YL and XC: data curation. YL, YG, CT, HL, and MH: writing—review and editing. All authors have read and agreed to the published version of the manuscript.

## Funding

This work was supported by the National Natural Science Foundation of China (no. 82271562); the Research and development plan of Zhejiang Province “Pioneer” and “Leading Goose” (no. 2022C03G2012959).

## Conflict of interest

The authors declare that the research was conducted in the absence of any commercial or financial relationships that could be construed as a potential conflict of interest.

## Publisher’s note

All claims expressed in this article are solely those of the authors and do not necessarily represent those of their affiliated organizations, or those of the publisher, the editors and the reviewers. Any product that may be evaluated in this article, or claim that may be made by its manufacturer, is not guaranteed or endorsed by the publisher.
